# An update on the epidemiological situation of spotted fever in Brazil

**DOI:** 10.1186/s40409-016-0077-4

**Published:** 2016-08-22

**Authors:** Stefan Vilges de Oliveira, Jessica Noronha Guimarães, Guilherme Carneiro Reckziegel, Bidiah Mariano da Costa Neves, Keline Medeiros de Araújo-Vilges, Lidsy Ximenes Fonseca, Fernanda Voietta Pinna, Simone Valéria Costa Pereira, Eduardo Pacheco de Caldas, Gilberto Salles Gazeta, Rodrigo Gurgel-Gonçalves

**Affiliations:** 1 Secretariat of Health Surveillance, Brazilian Ministry of Health, Brasília, DF Brazil; 2 Graduate Program in Tropical Medicine, University of Brasília, Brasília, DF Brazil; 3 National Reference Laboratory of Vectors of Rickettsioses, Oswaldo Cruz foundation, Rio de Janeiro, RJ Brazil; 4 Graduate Program in Infectious and Parasitic Diseases, School of Veterinary Medicine, University of Brasília, Brasília, DF Brazil; 5 Laboratory of Medical Parasitology and Vector Biology, School of Medicine, University of Brasília, Brasília, DF Brazil

**Keywords:** Rickettsial diseases, Epidemiology, Information system, Tick-borne disease

## Abstract

**Background:**

Spotted fever is a tick-borne rickettsial disease. In Brazil, its notification to the Ministry of Health is compulsory. Since 2007, cases of spotted fever have been integrated to the Notifiable Diseases Information System, and epidemiological analyzes are part of the routines on surveillance programs.

**Methods:**

This descriptive study updates epidemiological information on cases of spotted fever registered in Brazil between 2007 and 2015.

**Results:**

In Brazil, 17,117 suspected cases of the disease were reported and 1,245 were confirmed in 12 states, mainly in São Paulo (550, 44.2 %) and Santa Catarina (276, 22.2 %). No geographic information was registered for 132 cases (10.6 %). Most of the infected people were men (70.9 %), mainly in rural areas (539, 43.3 %), who had contact with ticks (72.7 %). A higher number of suspected cases were registered between 2011 and 2015, but the number of confirmed cases and the incidence were relatively low. Moreover, 411 deaths were registered between 2007 and 2015, mainly in the southeastern region of the country, where the case-fatality rate was 55 %. Lack of proper filling of important fields of notification forms was also observed.

**Conclusions:**

The results showed expansion of suspected cases of spotted fever and high case-fatality rates, which could be related to diagnostic difficulties and lack of prompt treatment. These factors may comprise limitations to the epidemiological surveillance system in Brazil, hence improvement of notification and investigation are crucial to reduce morbidity and mortality due to spotted fever in Brazil.

## Background

Spotted fever (SF) is an infection caused by gram-negative, obligate intracellular bacteria of the genus *Rickettsia*. The main agent of SF in Brazil is *Rickettsia rickettsii*. Nevertheless, other species of *Rickettsia* associated or not to SF clinical cases have been registered in several regions of the country [1–3]. *Amblyomma* ticks – such as *A. sculptum*, *A. aureolatum* and *A. ovale –* are the main vectors and their hosts (usually horses, capybaras and canines) are nonspecific, therefore, they may also parasitize humans [4, 5]. *Rickettsia rickettsii* SF is considered the most lethal rickettsial disease, and the maculopapular rash, its characteristic clinical sign, typically appears on the fifth day of infection [6]. However, it is not present in all cases. Rash absence interferes with the differential diagnosis, and *R. rickettsii* SF may be confused with other diseases including leptospirosis, dengue fever, ehrlichiosis, viral rash diseases, other rickettsial related diseases from typhus group, etc. [6–8]. Moreover, *Rickettsia parkeri* strain Atlantic rainforest is present in several regions of Brazil, causing a milder spotted fever with characteristic signs (inoculation eschar and lymphadenopathy) [1, 2].

SF has been observed in Brazil since 1929, but only in 2001 it was included in the list of diseases of compulsory notification (DCN) from the Ministry of Health (MH) [9, 10]. Since 2014, it has become a disease of compulsory and immediate notification. Consequently, health professionals are held responsible for reporting suspected and/or confirmed cases to the municipality, state and the Brazilian Ministry of Health within 24 h. Cases of SF are registered into the Notifiable Diseases Information System (SINAN), which aims to collect, gather and disseminate data on this disease [11]. Currently, SF is considered an emerging disease in Brazil due to the increase in diagnosed cases and expansion of occurrence areas. However, it is still little known by health professionals and the general public [12].

Clinical, epidemiological and laboratory aspects must be considered to confirm cases of SF. Epidemiological history of suspected cases helps and may lead to a timely diagnosis supported by laboratory tests [13, 14]. Epidemiological studies are necessary to evaluate the disease behavior over space and time and may help surveillance programs in Brazil [15]. Thus, this study updates epidemiological information on cases of SF registered in Brazil between 2007 and 2015.

## Methods

A descriptive epidemiological study was performed based on cases of spotted fever notified by investigation forms (IF) available in SINAN between 2007 and 2015. The database was accessed on March 14th, 2016. Duplicate or inconsistent information (out of the case definition proposed by the MH) were excluded from the analysis.

Cases were considered suspected whenever the individual presented fever, headache, myalgia and history of tick bites, and/or contact with domestic and/or wild animals, and/or had been to SF known transmission areas in the last 15 days, and/or presented maculopapular rash or hemorrhagic manifestations [13, 14]. Cases were confirmed when signs, symptoms and epidemiological history matched the suspect case definition and when infection with *Rickettsia* from the spotted fever group (SFG) was established [13, 14].

The laboratory testing recommended for SF by the MH consists of serological evidence of a fourfold change in immunoglobulin G (IgG)-specific antibody titers reactive to *Rickettsia rickettsia* SF group antigens by indirect immunofluorescence assay (IFA) between paired serum samples (one taken in the first week of illness and a second 2–4 weeks later). Moreover, it is also recommended the detection of *R. rickettsia* or other SF group DNA in a clinical sample via amplification of a specific target by PCR assay, or demonstration of SF group antigens in a biopsy or autopsy sample by immunohistochemistry, as well as isolation of *R. rickettsia* or other SF group *Rickettsia* from a clinical sample in cell culture.

Variables associated with confirmed cases were analyzed according to the following classification [16]:

- General data of patients about their origin – region of Brazil, federative unit (FU) of notification and municipality of infection.
- Individual data – age, gender and ethnic group.
- Clinical data – main signs and symptoms.
- Epidemiological data – related specifically to exposure risk.
- Treatment – if there was hospitalization, dates of admission and discharge.
- Specific laboratory data – if there were laboratory tests.
- Conclusion data – evolution of cases, confirmation or disposal criterion, location and characteristics of the probable place of infection.

The annual incidence rates were calculated employing the total population per studied year obtained from the Brazilian Institute of Geography and Statistics (IBGE) [17]. The absolute number of deaths and confirmed cases of SF were used to calculate the annual case-fatality rate. Average coefficients of incidence of SF were calculated for municipalities and states (cases/100,000 inhabitants per year) [17].

A proportion between missing/blank records and total filled fields was carried out for the observed variables to assess the completeness of forms. It was based on the inclusion criteria of the confirmed cases, involving mandatory (dates of notification, investigation start, first symptoms and case closing), essential (dates of hospitalization and development of the case) and conditioned variables (dates of hospitalization, discharge and death).

SINAN parameters were used to evaluate the completeness of data: excellent > 90 %, regular = 70–89 % and bad < 70 % of the filled fields. Timeliness was calculated using the difference in days between the intervals between the following dates: notification and investigation, notification and closing, the first symptoms and hospitalization, the first symptoms and discharge, the first symptoms and death.

The software TabWin 32 version 3.6 [18] was used for data tabulation, Microsoft Office Excel 2010 for charts and graphs, and TerraView version 3.2.0.1 for maps [19]. Secondary data was used in accordance with the National Health Council Resolution 466/2012 and does not cover information that could identify individuals registered in the information system [20].

## Results

**Fig. 1:**
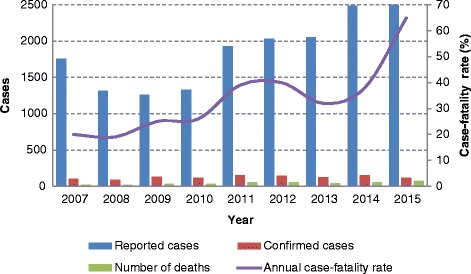
Absolute number of reported cases, confirmed ones and deaths due to spotted fever and annual case-fatality rate between 2007 and 2015 in Brazil

Between 2007 and 2015, 17,117 suspected cases of spotted fever were reported and 1,245 were confirmed in 12 states: São Paulo (*n* = 550, 44.2 %), Santa Catarina (*n* = 276, 22.2 %), Minas Gerais (*n* = 104, 8.5 %), Rio de Janeiro (*n* = 90, 7.2 %), Espírito Santo (*n* = 32, 2.5 %), Paraná (*n* = 25, 2.0 %), Ceará (*n* = 11, 0.9 %), Rio Grande do Sul (*n* = 9, 0.7 %), Goiás (*n* = 7, 0.5 %), Bahia (*n* = 4, 0.3 %), Mato Grosso do Sul (*n* = 4, 0.3 %) and Rondônia (*n* = 1, 0.1 %). No geographic information was registered for 132 cases of SF (10.6 %). Between 2007 and 2009, the number of reported cases decreased, but in the following years, there was an increase in the number of notifications (Fig. 1).

**Fig. 2:**
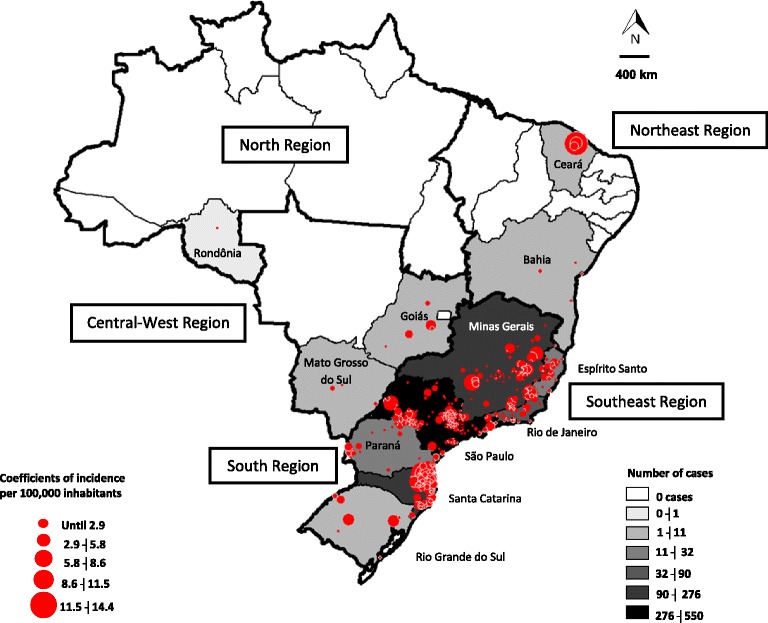
Geographical distribution of confirmed cases of spotted fever by federative unit (FU) and average incidence rate in affected municipalities between 2007 and 2015

The highest incidence rates were observed in Santa Catarina (0.48/100,000 inhabitants) and São Paulo (0.14/100,000 inhabitants) states. Higher incidences were also observed in the municipalities of Rodeio, Corupá and Luiz Alves (14.3, 13.9, and 13.8 respectively) (Fig. 2). Most infected people were white males between 20 and 64 years of age, from rural areas and who had contact with ticks (Table 1). Deaths were more frequent in males (79.1 %) than females (Table 1). Females had home as the primary site of infection acquisition (210/321, 65.4 %) while males probably acquired infection during leisure activities (239/779, 30.6 %).

**Table 1:** Absolute number and percentage of confirmed spotted fever cases and deaths regarding probable area of infection, relation to work, exposure to risk environments and hospitalization, between 2007 and 2015 in Brazil

	Cases		Deaths	
	N	%	N	%
Gender				
*Male*				
<1 year	4	0.45	2	0.61
1–4 years	20	2.26	8	2.46
5–9 years	49	5.54	15	4.61
10–14 years	46	5.20	18	5.53
15–19 years	56	6.34	22	6.76
20–49 years	445	50.39	151	46.46
50–64 years	209	23.66	82	25.23
65–79 years	53	6	27	8.30
>80 years	1	0.11	0	0
*Female*				
<1 year	5	1.38	1	1.16
1–4 years	27	7.45	6	6.97
5–9 years	27	7.45	10	11.62
10–14 years	31	8.56	12	13.95
15–19 years	20	5.52	5	5.81
20–49 years	183	50.55	37	43.02
50–64 years	53	14.64	12	13.95
65–79 years	15	4.14	3	3.48
>80 years	1	0.27	0	0
Ethnic Group				
White	816	65.54	220	53.53
Black	56	4.5	32	7.79
Asian	7	0.56	0	0
Multiracial	215	17.27	90	21.9
Indigenous	4	0.32	1	0.24
Missing/blank	147	11.81	68	16.55
Probable area of infection				
Rural	539	43.29	146	35.52
Urban	407	32.69	139	33.82
Periurban	173	13.90	70	17.03
Missing/Blank	126	10.12	56	13.63
Work-related transmission				
Yes	213	17.11	63	15.33
No	860	69.08	264	64.23
Missing/blank	182	14.62	84	20.44
Exposure to risk environments				
Yes	831	66.75	254	61.80
No	255	20.48	64	15.57
Missing/blank	159	12.77	93	22.63
Hospitalization				
North	9	0.72	1	0.24
Northeast	1	0.08	0	0
Southeast	615	49.39	336	81.75
South	37	2.97	4	0.97
Central-West	6	0.48	1	0.24
Missing/blank	92	7.38	49	11.92

The majority of SF patients had visited environments such as forests, rivers and waterfalls (66.7 %) and were exposed to ticks (72.7 %). Other animals such as capybaras (15.6 %), dogs and cats (42.4 %), cattle (17.2 %) and horses (17.4 %) were also reported in the investigation forms of confirmed cases. A higher incidence rate was observed between September and November (41 %). A total of 207 cases of SF (16.6 %) were registered in October.

A total of 411 deaths were registered resulting in an overall case-fatality rate of 33 %. The southeastern region showed the highest case-fatality rate (55 %). An increase of 57.9 % of deaths was observed in the last years (Fig. 1). Among confirmed cases, 760 (61 %) were hospitalized, of which 391 died, representing a case-fatality rate of 51.4 % (Table 1).

**Fig. 3:**
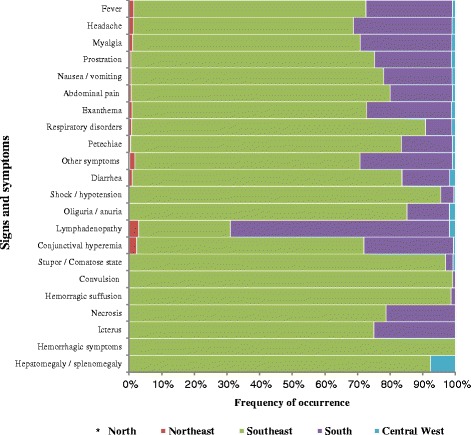
Main spotted fever signs and symptoms reported to the Notifiable Diseases Information System, by Brazilian region, between 2007 and 2015. *In the North region only one case was reported that presented the following symptoms: nausea/vomiting, prostration, myalgia, fever and headache

Laboratory criteria was the most used in the study period (*n* = 1,223, 90.2 %) then clinical and epidemiological 103 (8.2 %). The main SF signs and symptoms reported were fever, headache, myalgia, prostration and nausea/vomiting that had a different distribution among Brazilian regions (Fig. 3).

Although most information was available in the notification forms, the discharge date and date of death were the questions less answered (Table 2). Hospitalization date and progression of cases received more responses and were rated as excellent.

**Table 2:** Completeness of notification forms for spotted fever in Brazil (2007–2015) concerning dates of notification of the main variables, timeliness of notification and reporting of the first symptoms

Field	Field completeness	
	N	(%)
Date of notification	1245	100
First symptoms	1245	100
Investigation start	1245	100
Hospitalization	1238	99.43
Admission	750	60.24
Discharge	368	29.55
Course of the case	1197	96.14
Death^a^	424	34.05
Closing	1245	100
Timeliness – notification	Media (days)	Median (days)
Investigation date	16.57	6
Closing date	53.36	40
Timeliness – reporting of first symptoms	Media (days)	Median (days)
Admission date	7.02	4
Discharge date	16.38	8
Death^a^	12.02	6

^a^Death due to spotted fever

## Discussion

An increase in the number of suspected cases of SF was observed between 2007 and 2015; however, the number of confirmed cases presented low variation. High case-fatality rates were registered in southeastern Brazil. South and Southeast regions had the highest incidence rates, with most cases being reported in São Paulo and Santa Catarina, a finding corroborated by Barros-Silva et al. [21]. Moreover, the present study shows the first cases of SF in the states of Mato Grosso do Sul and Rondônia, where *Rickettsia* species were already found in ticks [22, 23]. These results show an expansion of SF in Brazil.

The incorporation of molecular diagnostic techniques by the MH in 2011 increased the number of deaths attributable to SF in Brazil, which could explain the high case-fatality rates registered in the present study [13, 14]. Such techniques allowed the identification of suspected cases that progressed to death without diagnosis using a unique sample. The present work also included records of deaths in southern Brazil, in transition areas within the state of São Paulo, which indicates the possible occurrence of *Rickettsia rickettsia*. This situation comprises a threat to public health and highlights the need for research on the strains occurring in these regions. Increase in the number of notifications is a result of efforts promoted by the MH, which is developing continuous training processes and educational activities aimed at structuring a network for surveillance of spotted fever and other rickettsial diseases in the country [24].

In the United States of America, a public health system (TickNET) promotes surveillance, research, education and prevention of tick-borne diseases. Nevertheless, notification of cases is not mandatory. In that country, cases are reported under a category called spotted fever rickettsiosis [25–27]. Since the 1930s, countries in the Americas, such as Colombia and Mexico, began to register focal cases of rickettsial diseases as *fiebre de tobia* and *fiebre manchada*, respectively. However, these records are collected by research centers and public information is not provided.

The present findings show that most of the confirmed victims were males, which is in agreement with international reports of the Centers for Disease Control and Prevention and the European Center for Disease Prevention and Control [26, 28]. In Brazil, men were infected in leisure activities whereas women become infected in household chores [29]. The most affected ethnic group in Brazil was white people, especially in the Southeast and South. However, rash is not easily seen in black complexion and this could be hindering the diagnosis of SF cases in this ethnic group [30].

The highest incidence was observed in rural areas in individuals who visit forest areas, rivers and waterfalls. Souza et al. [31] reported a high SF incidence in urban areas in São Paulo. The same study also suggested that SF transmission is related to the existence of a dirty pasture environment where *A. sculptum* ticks and capybaras are present. Although the findings by Souza et al. [31] indicated no relationship between SF and work activities, there is an outbreak record mentioning cases of SF in an animal shelter, which suggests a risk of occupational exposure [32]. Such situation draws attention to the need of preventive practices for professionals engaged in activities that may pose risks for rickettsial infections [33].

Contact with ticks was registered in 72 % of investigation forms. *Amblyomma cajennense* tick complex has a wide distribution in Brazil [5]. Szabó et al. [4] emphasizes that although *Rhipicephalus sanguineus* tick is present throughout Brazil, its participation in epidemic cycles has not been definitively proven. Most cases of SF were registered in October, when a higher density of vectors (tick nymphs) is recorded, which may be associated with transmission to humans [34]. However, climate variations should be examined promptly within municipalities and Brazilian ecoregions.

The majority of the confirmed cases required hospitalization. Southeast was the region that had more records of cases and deaths. *R. rickettsii* have potential for the development of serious forms of the disease, with systemic disorders such as respiratory failure, oliguria, hemorrhagic manifestations, neurological changes and shock. High case-fatality rates may be associated with [29]:

- A low index of suspicion per health care professionals.
- Lack of knowledge on the clinical features of the disease, which is often confused with other infections that have similar symptoms such as dengue and leptospirosis.
- Eco-epidemiological aspects associated with the risk of infection.
- Risk factors for exposure to the vector in transmission areas of the disease.

In southern Brazil, the disease is milder and most cases do not require hospitalization. This fact may be associated with infections caused by *R. parkeri* – an Atlantic rainforest strain that leads to a benign evolution [35]. However, in the north of Paraná, fatal cases of the disease provoked by *R. rickettsii* were observed [16].

Laboratory tests were used for confirmation of the majority of the cases. The most commonly used is the serologic one that verifies IgM and IgG titers without specifically checking the species of the etiologic agent, which is one of the current limitations of laboratory diagnosis in Brazil. According to Labruna et al. [3], there are different species of *Rickettsia* of the spotted fever group in Brazil (*Rickettsia rickettsii*, *R. parkeri*, *R. felis*, *R. rhipicephali*, *R. bellii*, *R. typhi*, *R. amblyommii*, *R. andeane* and *R. monteiroi*), many of those with unknown pathogenicity.

The most frequent clinical signs were fever, headache and myalgia. The presence of rash, an important sign of the disease, was recorded in less than 40 % of the confirmed cases. It is necessary to assess the current procedures and to consider regional particularities, thus seeking to identify signs, symptoms and epidemiological characteristics that may be related to the *Rickettsia* species. SF caused by *R. rickettsii* evolves rapidly and without the correct and timely medical interventions, clinical cases can develop to severe clinical forms, such as icterus, seizures, hemorrhagic suffusion and extremity necrosis. Further studies should evaluate the burden of SF, considering the impact of deaths of children, young people and adults in working age, as well as amputations resulting from necrotic processes and other sequels. All these injuries generate loss of life years, disability, economic costs to the health system and social security, sick leaves and pensions.

In 2013, the treatment protocol for cases of spotted fever was revised in Brazil, adding new recommendations, with emphasis on use of doxycycline as the first choice medication, regardless of age. The Ministry of Health of Brazil started to provide these drugs in strategic places in endemic areas [36]. Future evaluations of the effectiveness of these new treatment guidelines on case-fatality rates will be required.

The main limitation of this study is the underreporting of cases to epidemiological surveillance systems. The addition of other databases of the Brazilian health system in future analysis – such as those from hospitals, laboratories and mortality information system – would certainly quantify new cases of SF. Low completeness may also interfere with results of studies based on SINAN databases. Regarding confirmed cases, there was complete filling of the notification dates, early symptoms, early research and closing date of the cases. These fields are crucial for understanding the quality of health care in cases of SF. Timeliness – which was analyzed between admission, discharge and death – demonstrated a rapid clinical course and the need to rapidly identify cases and take the appropriate treatment in order to reduce case-fatality rate.

## Conclusions

The results showed expansion of suspected cases of spotted fever and high case-fatality rates, which could be related to diagnostic difficulties and lack of prompt treatment. These factors may be limitations of the SF Epidemiological Surveillance System in Brazil, hence improvement of notification and investigation are crucial for to reduce morbidity and mortality due to SF in Brazil.
